# An Efficacious Endometrial Sampler for Screening Endometrial Cancer

**DOI:** 10.3389/fonc.2019.00067

**Published:** 2019-02-19

**Authors:** Lu Han, Jiang Du, Lanbo Zhao, Chao Sun, Qi Wang, Xiaoqian Tuo, Huilian Hou, Yu Liu, Qing Wang, Qurat Ulain, Shulan Lv, Guanjun Zhang, Qing Song, Qiling Li

**Affiliations:** ^1^Center for Single-Cell Biology, First Affiliated Hospital, Xi'an Jiaotong University, Xi'an, China; ^2^Department of Obstetrics and Gynecology, First Affiliated Hospital, Xi'an Jiaotong University, Xi'an, China; ^3^Department of Gynecology and Obstetrics, Shaanxi Provincial People's Hospital, Xi'an, China; ^4^Guipei 77, Health Science Center, Xi'an Jiaotong University, Xi'an, China; ^5^Department of Pathology, First Affiliated Hospital, Xi'an Jiaotong University, Xi'an, China; ^6^Cardiovascular Research Institute, Morehouse School of Medicine, Atlanta, GA, United States

**Keywords:** endometrial cancer, endometrial sampler, cytology, histopathology, screening

## Abstract

Recently, the research on early detection of precancerous change and endometrial carcinoma has been focusing on minimally invasive procedures for screening. On this basis, we aim to verify the feasibility of endometrial samplers for screening endometrial cancer using Li Brush. We recruited patients undergoing hysterectomy for different diseases from the Inpatient Department of the Department of Obstetrics and Gynecology. Before surgery, endometrial cells were collected by Li Brush. The cytopathologic diagnosis from Li Brush and the histopathologic diagnosis from hysterectomy in the same patient were compared to calculate sensitivity (Se), specificity (Sp), false-negative rate (FNR), false-positive rate (FPR), positive predictive value (PV+) %, and negative predictive value (PV-). The research enrolled 293 women into this self-controlled trial. According to the hypothesis test of paired four lattices, we obtained the following indicators: Se 92.73, Sp 98.15, FNR 7.27, FPR 1.85, PV+92.73, and PV−98.15%. The endometrial sampler Li Brush is an efficacious instrument for screening endometrial cancer.

## Introduction

The morbidity and mortality of endometrial carcinoma is on the rise around the world in recent years. It has been the most common gynecologic malignancy in some developed countries such as Japan and US and ranked second in many developing countries (1, 2). The cancer-related costs are increasing significantly, constituting a challenge for social economics and female health. Efforts focusing on primary and secondary prevention remain central to the global charge to reduce the incidence of cancer and avoid one-third to one-half of cancer deaths (2, 3). With developing morbidity of endometrial cancer around the world, early detection and diagnosis would undoubtedly become the most important part. For endometrial carcinoma, the 5-year survival rate gradually decreases with the development of the stages. Eighty percent of the patients diagnosed with endometrial cancer are in stage I, with a 5-year survival rate of >95% (4). Endometrial atypical hyperplasia is considered to be the precancerous lesions of endometrial cancer. Thirty percent of atypical hyperplasia will develop into cancer a long time in the future; thus, we have the opportunity to screen for endometrial cancer within this long time period (5). The aim of screening is to detect endometrial atypical hyperplasia and the early stages of endometrial cancer. The ability to save lives would mean great social significance and economic benefits.

Endometrial carcinoma is a type of epitheliogenic malignant tumor that originates from the endometrium. As one of three major malignant tumors of the female reproductive system, the average onset age of endometrial carcinomas is 63 years, and > 90% occur in women above 50 years of age, and ~4% occur in women younger than 40 years of age (4). Risk factors for endometrial cancer include early menarche (6), late menopause, nulliparity, Lynch syndrome (7), diabetes (8, 9), obesity (10), hypertension, estrogen, and tamoxifen treatment after menopause (11, 12), a family history of endometrial cancer or breast cancer (13), and polycystic ovary syndrome (14).

**Figure 1:**
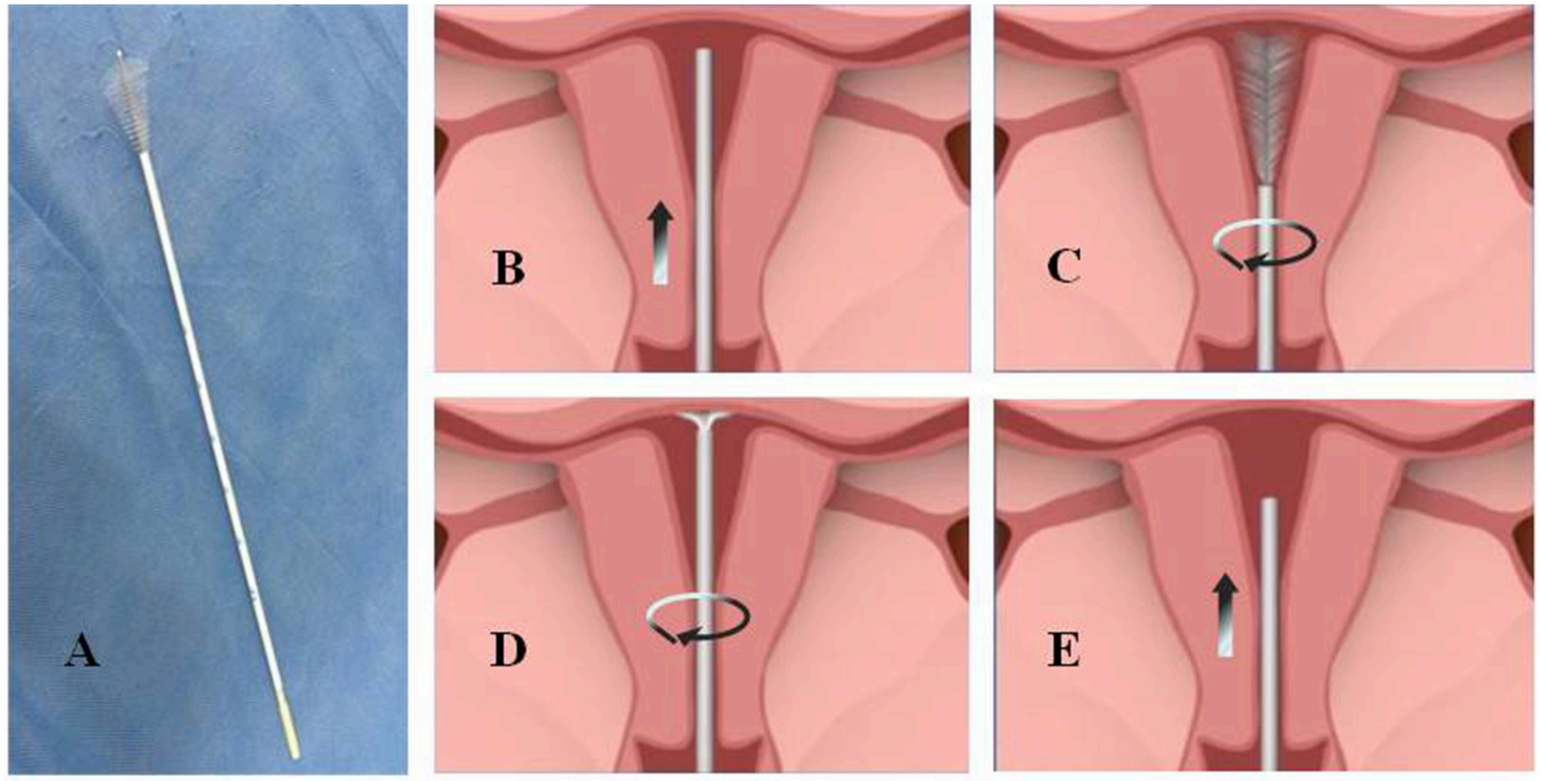
The physical map and sampling procedure of endometrial samples using Li Brush**. (A)** a photo of Li Brush; **(B)** protect the brush head by placing it into the drivepipe and put sampler into the fundus of the uterus; **(C)** withdraw the drivepipe approximately 5 mm to show the brush and rotate the handle in 5 to 10 complete circles to gather cells of the uterine corpus; **(D)** advance the drivepipe 3 mm and rotate the handle again to gather cells of the uterine fundus; **(E)** cover the head with the casing and withdraw the brush from the uterine cavity.

Because of the rising morbidity, the window period, and an explicit screening population, endometrial cancer screening is feasible. Until now, histopathology with dilatation and curettage (D&C) with or without hysteroscopy and surgery has been the gold standard for the diagnosis of endometrial carcinoma and precancerous lesions (15). However, the injury and discomfort caused by D&C have influenced its widespread use for screening. In recent years, more and more non-invasive endometrial devices have been invented and proposed for screening endometrial cancer, such as Pipelle, which was found to have an 86% sensitivity in one study (16); Tao Brush, which was found to have a 95.5% sensitivity (16); and SAP-1, which was found to have a 73% sensitivity, 95.8% specificity, 75% positive predictive value, and 95.3% negative predictive value (17). However, no available, specific, and effective screening method could be applied popularly for women until now. In this study, we compared the cytopathologic diagnosis of the Li Brush (Figure 1A, Xi'an Meijiajia Medical Co. 20152660054) with the histopathologic diagnosis of hysterectomy to evaluate the feasibility of the endometrial samplers for screening endometrial cancer.

## Materials and Methods

### Patients and Study Procedures

From January 2015 to July 2016, we recruited patients undergoing hysterectomy because of different diseases from the Inpatient Department (IPD) of the Department of Obstetrics and Gynecology. Patients in the IPD were excluded if they had already been diagnosed with pregnancy, acute inflammation of the genital system besides atrophic vaginitis, endogenous cervical carcinoma, dysfunction of blood coagulation, and other hematologic diseases that might influence coagulation function. Women with a body temperature >37.5°C in two subsequent measurements in 1 day were also ruled out.

According to the following procedures, we collected endometrium specimens using the Li Brush and obtained the cytopathologic diagnosis from the Department of Pathology before surgery. First, the patients were placed in the lithotomy position, and the conventional perineal and vaginal disinfection were performed after emptying the bladder. Second, the uterine cervix was exposed by vaginal speculum and the uterine depth was detected with uterine probe. After the brush head was hidden in the drivepipe, the sampler was put into the fundus of uterus (Figure 1B). Then, the drivepipe was drawn out ~5 mm to show the brush, and the handle was rotated 5–10 complete circles to gather cells of the uterine corpus (Figure 1C). Third, the drivepipe was advanced 3 mm and the handle was again rotated to gather cells from the uterine fundus (Figure 1D). Finally, the brush was removed from uterine cavity after protecting the brush head under the casing (Figure 1E). When sampling was complete, the brush head was placed into the preservation solution and shaken several times to release the cells into the solution.

The cell specimens were prepared for testing with a liquid-based cytologic test, and the tissues were embedded in paraffin and cut into cross-sections. Both cell and tissue specimens were stained using hematoxylin and eosin. After concealing identity information, all the samples were sent to the Department of Pathology of First Affiliated Hospital of Xi'an Jiaotong University and randomly diagnosed by two independent professors. The diagnoses of histology and cytology were independently conducted. According to the method of sample size calculation in the diagnosis experiment (18) and using the sensitivity of SAP-1 brush (4, 17) to predict the sensitivity of Li Brush, a minimum of 113 cases were required for the study. The self-control method was used in this study. The histopathologic diagnosis from the hysterectomy was defined as the standard. The outcomes were obtained by comparing the cytopathologic and histopathologic results of the same individuals.

The study was conducted in accordance with the Declaration of Helsinki, and the study protocol was approved by the Ethics Committee of the First Affiliated Hospital of Xi'an Jiaotong University(XJTU1AHCR2014-007). All the patients involved in the research were sufficiently informed of the content of the study and provided written informed consent.

### Data Collection

For all eligible patients, the following information was collected: age, age at menarche, last menstrual period or menopausal age, childbearing history, endometrial thickness, tumor history, smoking history, with or without hormone replacement, and history of other diseases such as hypertension and diabetes. The data were used to determine sampling satisfaction, cytopathologic diagnosis, and histopathologic diagnosis.

### Definition of Outcomes

According to the International Society of Gynecological Pathologists, the histopathologic diagnoses included the following: proliferative endometrium, secretory endometrium, atrophic endometrium, mixed endometrium, and simple hyperplasia including cystic glandular hyperplasia, complex hyperplasia defined as adenomatous hyperplasia without atypia, endometrial atypical hyperplasia, and endometrial carcinoma. The cytopathologic diagnoses were classified into seven categories, as follows: proliferative endometrial cells, secretory endometrial cells, atrophic endometrial cells, mixed endometrial cells, endometrial hyperplasia cells, endometrial atypical cells, and endometrial cancer cells. Positive results were defined as endometrial carcinoma, endometrial cancer cells, endometrial atypical hyperplasia, and endometrial atypical cells. Other categories were defined as negative results. When both cellular and histionic diagnostic results were positive, it was judged as true positive; if both were negative, it was judged as true negative. If the cytopathologic result was positive and the histopathologic result was negative, it was judged as false positive; if the cytopathologic result was negative and the histopathologic result was positive, it was judged as false negative. Consistent outcome was when the cytopathologic and histopathologic diagnoses were both positive or both negative; otherwise, the outcomes were considered inconsistent. The sampling satisfaction was reflected in a sufficient number of cells and the correct location.

### Statistical Analysis

Using the hypothesis test of paired four lattices, the following indicators were calculated: sensitivity (Se), false-negative rate (FNR), specificity (Sp), false-positive rate (FPR), positive predictive value (PV+), and negative predictive value (PV-). The differences of endometrial histopathology and cytopathology using Li Brush in the diagnosis of endometriosis were evaluated by the calculation of *P*-value. If *P* < 0.05, the difference was statistically significant. In contrast, if *P* > 0.05, the difference was not statistically significant.

## Results

### Patients

**Figure 2:**
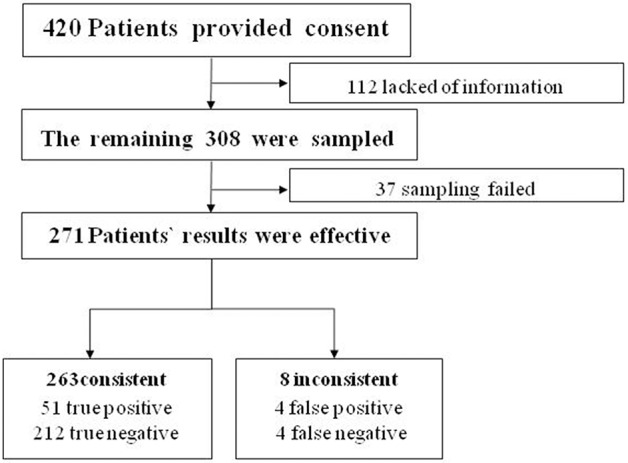
Diagram of study participants.

We aimed to collect a total of 420 patients from the IPD of the Department of Obstetrics and Gynecology. We ruled out 112 patients because of insufficient or incomplete information. The remaining 308 women completed the study (Table 1). Satisfactory endometrial cells were not obtained in 37 patients (Figure 2).

**Table 1:** Patient characteristics.

**Characteristics**	***n***
**SOURCE**	
IPD^a^	308
**AGE**	
< 40 years old	32
≥40 years old	276
**MENSTRUAL STATUS**	
Premenopausal	200
Postmenopausal	83
AUB^b^	6
**ENDOMETRIAL THICKNESS^c^**	
< 5 mm	24
≥5 mm	211
Intrauterine heterogeneity echo	5
Unclear display	3
**OTHER DISEASE**	
Ovarian cancer	3
Hypertension	7
Diabetes	4
Hormone replacement therapy	2

Some information of the patients is missing.

a IPD, Inpatient Department.

b AUB, Abnormal uterus bleeding.

c *Some patients were not examined by ultrasound, whose endometrial thickness is missing*.

### Pathologic Images

**Figure 3:**
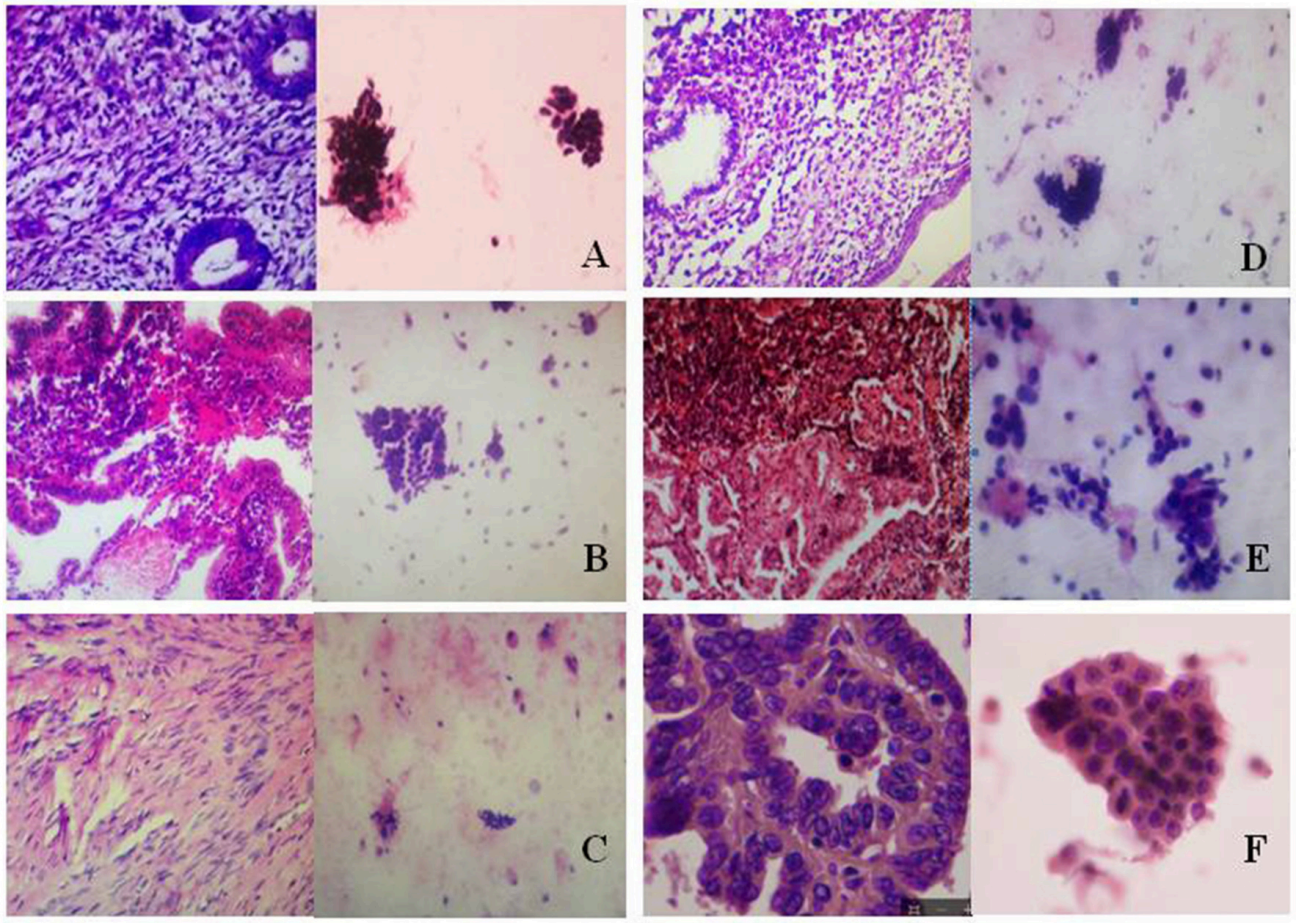
Histopathologic and cytopathologic images**. (A)** proliferative endometrium (Left: HE × 400) and proliferative endometrial cells (Right: HE × 100); **(B)** secretory endometrium (Left: HE × 10) and secretory endometrial cells (Right: HE × 10); **(C)** atrophic endometrium (Left: HE × 10) and atrophic endometrial cells (Right: HE × 10); **(D)** mixed endometrium (Left: HE × 10) and mixed endometrial cells (Right: HE × 10); **(E)**: endometrial atypical hyperplasia (Left: HE × 10) and endometrial atypical cells (Right: HE × 200); **(F)** endometrial carcinoma (Left: HE × 400) and endometrial cancer cells (Right: HE × 400).

Using pathological slide and microscopic camera technology, the following histrionic images were obtained: proliferative endometrium (Figure 3A, left), secretory endometrium (Figure 3B, left), atrophic endometrium (Figure 3C, left), mixed endometrium (Figure 3D, left), endometrial atypical hyperplasia (Figure 3E, left), and endometrial carcinoma (Figure 3F, left). The corresponding cytopathologic images were included: proliferative endometrial cells (Figure 3A, right), secretory endometrial cells (Figure 3B, right), atrophic endometrial cells (Figure 3C, right), mixed endometrial cells (Figure 3D, right), endometrial atypical cells (Figure 3E, right), and endometrial cancer cells (Figure 3F, right).

### Data Calculation

According to the hypothesis test of paired four lattices, there were 51 true-positive, 212 true-negative, 4 false-positive, and 4 false-negative cases. The following indices were obtained: Se 92.73, Sp 98.15, FNR 7.27, FPR 1.85, PV+ 92.73, and PV- 98.15%. The data showed that there were no significant difference between cytopathologic results from the Li Brush and histopathologic results from hysterectomy (χ^2^ = 0.125 < $\chi_{0.05}^{2}$, α = 0.05, *P* > 0.05).

**Figure 4:**
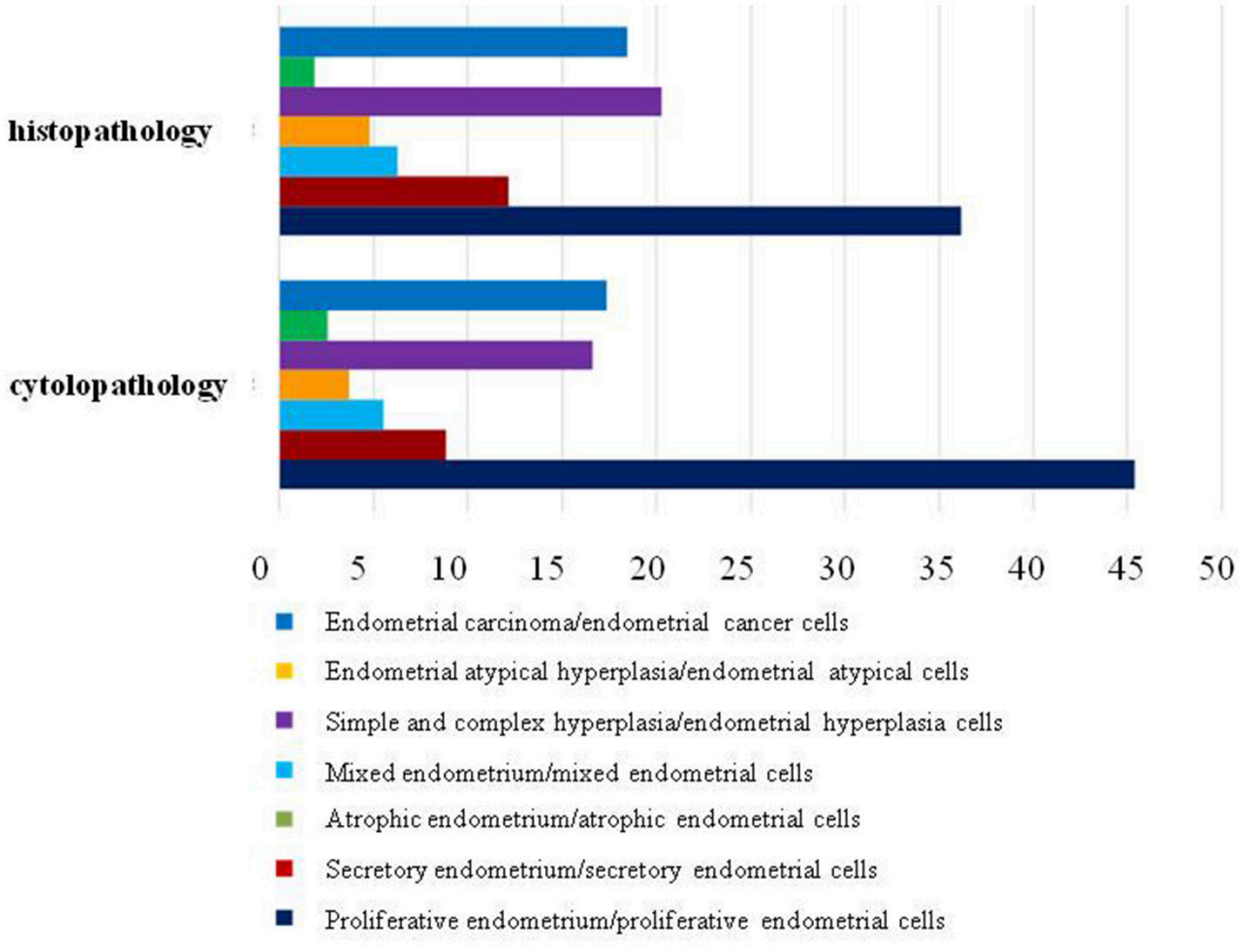
The proportions of histopathologic and cytologic diagnosis.

Furthermore, we compared the histopathologic results obtained from hysterectomy and the cytopathologic results of our samplers accurately to evaluate the feasibility of using Li Brush in the diagnosis of endometrial types. Through the statistics, there were 228 cases consistent and 34 cases inconsistent. In addition, 9 cases were diagnosed as endometrial simple hyperplasia with local polyps by hysterectomy and endometrial hyperplasia cells by our samplers. The overall degree of satisfaction and sensitivity of sampling were 87.02 and 87.63%, respectively (Table 2). The sensitivities of different types of endometrium were 85.88% for proliferative endometrium, 72.73% for secretory endometrium, 88.24% for atrophic endometrium, 83.33% for mixed endometrium, 94.64% for simple hyperplasia, 100% for complex hyperplasia, 80.00% for endometrial atypical hyperplasia, and 87.02% for endometrial carcinoma. Meanwhile, the results of cells and tissues were separately analyzed and compiled into a bar chart by composition ratios (Figure 4).

**Table 2:** The comparison of diagnosis between cytopathology and histopathology.

**Endometrial types**	**Cytopathology and histopathology**			**Total**	**Se(%)**	**Sampling satisfaction(%)**
	**Consistent**	**Inconsistent**	**Unsatisfied sampling**			
Proliferative endometrium	73	12	10	95	85.88	89.47
Secretory endometrium	24	9	5	38	72.73	86.84
Atrophic endometrium	15	2	4	21	88.24	80.95
Mixed endometrium	10	2	1	13	83.33	92.31
Simple hyperplasia	53	3	9	65	94.64	86.15
Complex hyperplasia	4	0	1	5	100.00	80.00
Atypical hyperplasia	4	1	1	6	80.00	83.33
Endometrial carcinoma	45	5^a^	6	56	90.00	89.29
Total	228	34	37	299^b^	87.02	87.63

Se, Sensitivity.

a There were 4 cases which the histopathological results were endometrial cancer but the cytopathologic results were proliferative endometrial or endometrial hyperplasia cells. One of this 5 cases which the histopathological result of was endometrial cancer but the cytopathologic result was endometrial atypical cells, so this case was considered to be true positive but inconsistent.

b *There were 9 of all 308 cases diagnosed endometrial simple hyperplasia with local polyp*.

## Discussion

Endometrial curetting or D&C have long been the standard diagnosis or treatment for evaluating suspicious endometrial lesions, especially in mainland China. Only patients who display symptoms that are geared to the indication of D&C, such as abnormal uterine bleeding, would refer themselves to medical help for diagnosis and treatment, which restricts the early diagnosis and treatment of endometrial carcinomas. Because only approximately 60% of curetting procedures can evaluate less than half of the uterine cavity, even when performed by the most experienced physician, the rate of false-negative results is high (19). Moreover, the pain and suffering caused by the procedure were not widely accepted patients. Despite its diagnostic value for patients who display symptoms, this method shows its deficiency as a screening procedure for endometrial lesions (20).

Recently, research on the early detection of endometrial carcinoma has been focusing on minimally invasive histopathologic and cytopathologic procedures (16, 21–24). Along with the improvement and widespread use of liquid-based preparation (LBP) of endometrial cell samples, direct cellular sampling has more commonly used as the primary screening procedure for endometrial lesions (25). Among the sampling techniques, endometrial brush cytology is minimally invasive, more economical, and more convenient compared with traditional diagnostic and curettage techniques. Therefore, it has already become a partly accepted method for the detection of endometrial lesions (26). The endometrial cytology by direct intrauterine sampling has a relatively high specificity and sensitivity for the diagnosis of endometrial cancer reported by some researchers (27). Related samplers have been studied, including histology samplers such as Pipelle (28), and cytology samplers, such as Tao brush (19) and SAP-1 sampler (4, 17), as well as Uterobrush (26). However, up to this point, we still required a more convenient, economical, and non-invasive tool to screen for endometrial cancer.

On that basis, we have invented a new endometrial sampler—Li Brush. Our sampler was awarded a utility model patent certificate from the State Patent Office (number: ZL.2014 2 0720056.8). The brush is made up of four parts: head,tube core, drivepipe, and hand shank (Figure 1A). The head is T shaped, which is close to the physiologic form of the uterine cavity. The fusiform brush allows easy access to the uterine cavity, fundus, and horn of the uterus. The elastic drivepipe works with the handle to protect the head from contamination of cervical cells. In addition, our samplers have other advantages, such as low cross-infection, good flexibility, less damage, low cost, and higher acceptability. In this study, we compared the diagnosis of cytology by the Li Brush with the diagnosis of histopathology by hysterectomy. A total of 271 cases were analyzed, with a sensitivity of 92.73% and a specificity of 98.15%. The proportions of the endometrial types were similar between histology and cytology. These findings showed that Li Brush will be able to play a role in the screening for endometrial cancer.

The two common causes of sampling failure using Li Brush were the inaccurate location of sampling and insufficient number of endometrial cells. The lower sampling satisfaction of atypical hyperplasia and complex hyperplasia are because of the limited sample size. The lower sampling satisfaction of atrophic endometrium is attributed to the atrophic cervix and the adhesion of cervix tube in post-menopausal women. To improve the sampling satisfaction, we considered whether using cervical clamps when using the brush could make it easier for the brush to smoothly enter the uterine cavity. We can also mechanically expand the cervical canal for patients with cervical adhesion or narrowing if necessary.

For endometrial polyps, Li Brush showed a high false-negative rate. According to histology, endometrial polyps are classified into four categories: non-functional glandular endometrial polyp, functional glandular endometrial polyp, adenomatoid polyp, and polyp with malignant transformation (5). Polyps consist of proliferating glands, blood vessels, and stroma because of the hyperplasia due to the high sensitivity of endometrium to estrogen (17). When brushing, we only tend to sample the superficial cells or glands of polyps, which makes the sample look like endometrial hyperplasia, and ignores its real structure. Reagan and Ng et al. Study pointed out that when sampled cells were out of the endometrial cycle, only a quarter of them were from polyp (5). Thus, histology often shows more sensitive results than cytology for the diagnosis of polyps.

Our study found that Li Brush will be able to be a reliable approach for screening endometrial cancer and may provide great benefits for the social economy and women's health. However, there are still some shortcomings to the technique, such as the false-negative rate for the diagnosis of endometrial polyps is high and the sampling satisfaction rates are not low enough. In the future, after obtaining more data, we hope to use the brush to diagnose the detailed pathologic types of endometrium.

## Author Contributions

LH, JD, and CS performed the clinic experiments. LZ, SL, and QL designed the endometrial sampler. HH and GZ performed the pathologic diagnosis. YL performed the pathological sections. HH also performed the pathological sections. QiW, QinW, and XT helped to perform the statistical analysis. LH and QL wrote the manuscript. QU revised the English grammar. QS helped to designed the experiments. QL conceived the study. All authors gave feedback and approved the final version of the manuscript.

### Conflict of Interest Statement

The authors declare that the research was conducted in the absence of any commercial or financial relationships that could be construed as a potential conflict of interest.
